# Determining women's awareness levels of gynecological cancer and their perceptions of breast cancer fatalism

**DOI:** 10.1590/1806-9282.20260194

**Published:** 2026-09-28

**Authors:** EsmaNur Özen, Fatma Ersin

**Affiliations:** 1 Harran University, Institute of Health Sciences – Şanlıurfa, Turkey.; 2 Harran University, Faculty of Health Sciences, Department of Public Health Nursing – Şanlıurfa, Turkey.

**Keywords:** Gynecological, Breast, Cancer, Awareness, Nursing

## Abstract

**OBJECTIVE:**

This study was conducted to determine women's awareness levels regarding gynecological cancers and their fatalistic perceptions regarding breast cancer.

**METHODS:**

This was a descriptive study conducted in a Family Health Centre District between October 2023 and August 2024. The study sample consisted of 333 women. A personal information form, Gynecological Cancer Awareness Scale, and breast cancer fatalism scale were used to collect data.

**RESULTS:**

The mean total score on the Gynecological Cancer Awareness Scale for the women participating in the study was 146.02±21.70, and the mean total score on the Breast Cancer Fatalism Scale was 7.40±2.61. Educational status, social security, obtaining information from healthcare professionals about gynecological cancers, and previous gynecological examination status of the participants explain 18.7% of the variance in the Gynecological Cancer Awareness Scale total score average. Additionally, age and educational status explain 13.6% of the variance in the Breast Cancer Fatalism Scale total score average.

**CONCLUSION:**

As a result, it can be said that women's awareness levels about gynecological cancers and their perception of breast cancer fatalism are above average. Therefore, education should be provided to increase awareness levels of gynecological cancers and reduce perceptions of breast cancer fatalism.

## INTRODUCTION

Gynecological cancers, defined as malignant diseases of the female reproductive organs, are one of the leading causes of morbidity and mortality in women after breast cancer^1,2^. Gynecological cancers account for 7.24% of all cancer cases^3^. It is estimated that nearly one-fifth of all cancers occurring in women are gynecological cancers^4^. According to Global Cancer Statistics 2022, 6.17% of the 240,013 people newly diagnosed with cancer in our country were diagnosed with gynecological cancer. Of those diagnosed with gynecological cancer, 4.45% died^5^. Awareness among women of the factors that may contribute to gynecological cancers, along with participation in early diagnosis and screening, reduces the risk of death and illness caused by cancer^6^. Awareness levels among women are crucial in preventing gynecological cancers^7^. There are only a limited number of studies examining women's awareness levels regarding gynecological cancers^2,8^, and these studies indicate that women's awareness levels are not high.

Although there are many factors affecting women's awareness, one of the important ones is fatalism. Individuals with fatalistic beliefs may think that there is no relationship between cancers and lifestyle and that early diagnosis and screening programs serve no purpose other than diagnosing the disease. This mindset may also lead to less use of early diagnosis services^9^.

Breast cancer is one of the cancers with a very high incidence in women. The fatalistic attitude toward breast cancer plays a very important role in the early diagnosis and treatment of breast cancer in women^10^. Individuals who adopt a fatalistic approach may oppose early diagnosis and screening programmes^11^. The literature reports that breast cancer fatalism has a negative impact on early diagnosis and screening behaviours^12^. The literature includes studies with low^13^ and high levels of breast cancer fatalism perception^14^.

In conclusion, it is evident that awareness levels and fatalistic perceptions play a significant role in the early detection of cancers observed in women. However, the limited number of studies on awareness levels regarding gynecological cancers highlights the need for further research in this area. The literature emphasizes the importance of determining the current situation in order to increase women's awareness levels regarding gynecological cancers. Furthermore, it is thought that women's awareness levels regarding gynecological cancers influence their fatalistic perceptions of breast cancer.

This study was conducted to determine women's awareness levels regarding gynecological cancers and their fatalistic perceptions regarding breast cancer.

### Research questions

What are women's levels of gynecological cancer awareness and breast cancer fatalism perceptions?

What factors influence women's awareness of gynecological cancer and their perception of breast cancer fatalism?

## METHODS

This descriptive study was conducted in the a Family Health Centre Region between October 2023 and August 2024.

In calculating the sample size of the research, the sample calculation method with a known population was used. The population size was taken as 2,497 women between the ages of 30–65, affiliated with a family health center; 95% confidence level (Z=1.96), 5% margin of error (d=0.05), and expected prevalence value p=0.50 were accepted. The minimum number of samples was found to be 333. Women who were not diagnosed with gynecological cancer and/or breast cancer and who had not had a hysterectomy were included in the study. During the sample selection process, the addresses of women who met the inclusion criteria were listed first. The addresses to be visited were then selected randomly using a table. The women were visited at their homes, the purpose of the study was explained to them, and they were given data collection forms. All of the women selected for the sample agreed to participate in the study.

There were no refusals. The data were collected by the researcher visiting the women in their homes and conducting face-to-face interviews lasting 15–20 min. The introductory information form, Gynecological Cancer Awareness Scale (GCAS), and Breast Cancer Fatalism Scale (BCFS) were used to collect the study data.

The Information Form was developed by researchers through a literature review^2,8,15,16^.

Gynecological Cancer Awareness Scale (GCAS) consists of 41 items and was developed by Dal and Ertem to determine the level of awareness of gynecological cancers among women aged 20–65 years^16^. The GCAS has four subscales (Awareness of Early Diagnosis and Information on Cancers, Awareness of Gynecological Cancer Risks, Awareness of Prevention of Gynecological Cancers, and Awareness of Routine Check-ups and Perception of Serious Illness in Gynecological Cancers). The Cronbach's alpha value of the scale is 0.944. The score obtained from the scale ranges from a minimum of 41 to a maximum of 205. There is a direct correlation between the scale score and the level of awareness^16^. In our study, the Cronbach's alpha value of the GCAS is 0.91.

Breast Cancer Fatalism Scale (BCFS) is a 15-item scale developed by Powe and revised by Mayo et al to 11 questions^17,18^. Its validity and reliability were established in Turkey by Ersin et al. The scale consists of 11 items, with a "Yes" response scoring 1 point and a "No" response scoring 0 points, resulting in a total score ranging from a minimum of 0 to a maximum of 11. Higher scores on the scale indicate greater fatalism^10^. In our study, the Cronbach's alpha value of the scale was 0.87.

Statistical analysis of data: Data analysis was performed using the SPSS 26.0 software package. The significance level was set at p<0.05. Descriptive statistics including frequency, percentage, and mean were used in the analysis of data. Additionally, t-tests for independent groups, Kruskal-Wallis analysis, Pearson correlation analysis, simple linear regression, and multiple linear regression analysis were performed. Skewness and kurtosis coefficients were considered in assessing the suitability of the normal distribution. Skewness and kurtosis coefficients were expected to be in the range of −1 to +1^19^.

Ethical principles of the research: Permission was obtained from the Health Directorate (dated 08/12/2023 and numbered E-49781372-799-231234173) and the a University Clinical Research Ethics Committee (dated 13.11.2023 and numbered 2023/21/39) to conduct the research. Informed voluntary consent was obtained from the participants. The study was conducted in accordance with the Helsinki Declaration and the country's ethical standards.

## RESULTS

The study found that 77.2% of women were aged between 30 and 49, 89.8% of women were married, 53.5% of women were illiterate, 51.4% of women had a poor income, 91% of women were unemployed, and 71.8% of women had no social security (Table 1).

**Table 1:** Distribution of women's sociodemographic characteristics.

Variables		Number (n)	Percent (%)
Age (years)			
	Aged 30 to 49	257	77.2
	50 years old and above	76	22.8
Marital status			
	Single	34	10.2
	Married	299	89.8
Educational status			
	Illiterate	178	53.5
	Literate	61	18.3
	Primary school	86	25.8
	Secondary school and above	8	2.4
Income level			
	Poor	171	51.4
	Average	154	46.2
	Good	8	2.4
Employment status			
	Yes	30	9.0
	No	303	91.0
Social security status			
	Yes	94	28.2
	No	239	71.8

About 14.7% of participants stated that they had a gynecological condition, while 32.4% of participants stated that they had not undergone a gynecological examination. The most frequently cited reasons for not having a gynecological examination were not feeling the need for one (50%), feeling embarrassed about having one (44.7%), and fear of the examination (38.6%). About 74.5% of women stated that they had heard of gynecological cancers, and 69.5% of women stated that they had heard about gynecological cancers from healthcare personnel. In addition, 4.5% of women stated that there was a history of gynecological cancer in their family, and 7.2% of women stated that they knew the symptoms of gynecological cancer.

A significant difference was found in the GCAS total score average among the women participating in the study according to their educational status (KW=10.079, p=0.018), income status (KW=6.966, p=0.031), and social security status (t=3.417, p=0.001). Furthermore, a significant difference was found in the mean total BCFS score according to the women's age (t=-4.934, p=0.000), educational status (KW=35.857, p=0.000), income status (KW=7.653, p=0.022), and social security status (t=2.686, p=0.007).

The mean total GCAS score for women was 146.02±21.70, and the mean total BCFS score was 7.40±2.61. GCAS Early Diagnosis and Information Awareness in Gynecological Cancers sub-dimension was 15.92±2.92, Gynecological Cancer Risks Awareness sub-dimension was 28.4±4.43, Gynecological Cancers Prevention Awareness sub-dimension was 15.51±3.63, and Routine Control and Serious Disease Perception in Gynecological Cancers sub-dimension was 86.85±18.37.

A very weak, negative, statistically significant correlation was found between participants’ GCAS scores and their BCFS scores (r=-.222, p=0.000) (Table 2).

**Table 2:** Correlation of mean scores of the Gynecological Cancers Awareness Scale and its subdimensions, and the Breast Cancer Fatalism Scale.

Gynecological Cancers Awareness Scale and subdimensions average scores	BCFS total score average	
	r	p
Early diagnosis and information awareness sub-dimension in gynecological cancers	-0.265	0.000
Awareness of gynecological cancer risks sub-dimension	-0.054	0.335
Awareness of prevention of gynecological cancers sub-scale	-0.170	0.002
Routine control and perception of serious illness in gynecological cancers sub-dimension	-0.212	0.000
GCAS total average score	-0.222	0.000

BCFS: Breast Cancer Fatalism Scale; GCAS: Gynecological Cancer Awareness Scale.

Educational status, social security, obtaining information from healthcare professionals about gynecological cancers, and previous gynecological examination status of the participants explain 18.7% of the variance in the GCAS total score average. Additionally, age and educational status explain 13.6% of the variance in the BCFS total score average (Table 3).

**Table 3:** Multiple lineage regression analysis between certain characteristics of women and their awareness of gynecological cancers and perceptions regarding fatalism in breast cancer.

Dependent variables	Model	Independent variables	B	Standard error	Standardized coefficient β	t	p	95%CI	
								Lower	Upper
Breast cancer fatalism	1	Age	0.833	0.349	0.130	2.384	0.018	0.146	1.521
		Educational status	-0.884	0.158	-0.300	-5.611	0.000	-1.194	-0.574
		Income status	-0.232	0.264	-0.047	-0.877	0.381	-0.751	0.288
		Social security	0.617	0.317	0.103	1.946	0.053	-0.007	1.241
(R=0.369, R^2^=0.136, R^2^ adjusted=0.126, F=12.950; p=0.000, Durbin Watson=1.781)									
Awareness level of gynecological cancers	2	Educational status	2.805	1.225	0.118	2.290	0.023	0.395	5.214
		Income status	2.944	2.074	0.074	1.419	0.157	-1.137	7.025
		Social security	-6.175	2.478	-0.128	-2.492	0.013	-11.050	-1.300
		Obtaining information from healthcare professionals about gynecological cancers	-5.603	2.208	-0.129	-2.538	0.012	-9.947	-1.260
		Previous gynecological examination status	-14.285	2.370	-0.308	-6.027	0.000	-18.948	-9.623
(R=0.432; R^2^=0.187; R^2^ adjusted=0.175, F=15.044; p=0.000; Durbin Watson=1.614)									

R: correlation coefficient, R^2^: coefficient determinant, ß: standardized coefficient; CI: confidence interval.

## DISCUSSION

The study found that the mean GCAS total score for women was 146.02±21.70. In the literature, GCAS total scores for women range from 148±25.71 to 160.31±22.42^16,20,21^. Considering that the highest possible average score on the scale is 205, it can be said that the level of awareness of gynecological cancers among women in this study is above average. In this study, the mean total score for gynecological cancer awareness among women was found to be 7.40±2.61. In one study in the literature, the mean total score for gynecological cancer awareness was 4.69±2.67^21^ and 6.00±3.51^15^ as stated. In this study, women's above-average fatalistic beliefs may be a hindering factor in implementing health behaviors.

Our study found that women's educational status affects the GCAS total score average. Previous studies also support this finding^8,20^, and it is noted that awareness of cervical cancer increases as the level of education rises^21-23^. Considering that education is considered important in raising awareness, this study shows that education explains only a small fraction of awareness. Our study shows that income status affects the average GCAS total score, and the literature indicates that there is a significant relationship between income status and GCAS scores^20^. High income levels among women may have facilitated their access to healthcare services due to their high awareness of gynecological cancer. Women with social security coverage were found to have significantly higher mean GCAS total scores. The literature supports this finding^24^. In this study, having social security may have made it easier for women to communicate with healthcare personnel and increased their awareness.

This study shows that women's age affects the average total BCFS score. A study has indicated that fatalistic attitudes increase with age^25^. As women age, their fatalistic beliefs about breast cancer increase, suggesting that women associate cancer with age. Our study found that women's fatalism regarding breast cancer decreased as their level of education increased, a finding supported by the literature^17^. The fact that highly educated women had lower levels of fatalism suggests that they are more aware of breast cancer. This study shows that women with higher incomes have lower breast cancer fatalism scores. Given that income status facilitates individuals’ access to healthcare services, this result obtained from the study is plausible.

According to the regression analysis results, educational status, social security status, prior gynecological examination, and receiving information about gynecological cancers from healthcare personnel explain a low-to-moderate level of variance in gynecological cancer awareness. It can be said that age and education level explain the variance in breast cancer fatalism at a low-to-medium level. The high variance that the models cannot explain suggests that other factors may be effective (cultural beliefs, religious attitudes, health literacy, social support, personal experiences, etc.). Considering that awareness and fatalism are important in the development of positive health behaviors, the results obtained from the study are thought to contribute to the literature and are the expected results. The models show that social security is not significant in terms of fatalism regarding breast cancer. This suggests that, due to women's socio-cultural structure, their sense of privacy is paramount. Furthermore, the perceived ineffectiveness of social security may stem from the collinearity with education. The fatalism scale treats fatalism as a one-dimensional and binary structure for each item. This binary approach may have caused the variance to appear in this way.

## CONCLUSION AND RECOMMENDATIONS

In this study, it can be said that both the level of awareness of gynecological cancers and the level of fatalism regarding breast cancer were above average. Educational status, social security status, prior gynecological examination, and receiving information about gynecological cancers from healthcare personnel explain 18.7% of the variance in gynecological cancer awareness. Additionally, age and educational status explain 13.6% of the variance in the BCFS total score average.

Health education programs designed to increase women's awareness levels and reduce fatalism tendencies should be planned, implemented, and maintained by nurses in primary healthcare institutions. Furthermore, studies examining women's gynecological cancer awareness and breast cancer fatalism levels should be planned with a larger sample size.

### Limitations

Our study could be conducted with a larger sample size. The results of this study can only be generalized to women aged 30–65 years living in the a family health center area. The study results also show that most of the women are disadvantaged (many are illiterate, lack social security, and are unemployed). Therefore, we cannot generalize the study results to all women. The high rate of illiteracy may have affected the understanding of the data collection tools.

## Data Availability

The datasets generated and/or analyzed during the current study are available from the corresponding author upon reasonable request.
